# Ambient air pollution and consumer spending: Evidence from Spain

**DOI:** 10.1371/journal.pone.0292245

**Published:** 2024-01-24

**Authors:** John Brandt, Nihit Goyal, Matthew Moroney, Sophie Janaskie, Angel Hsu

**Affiliations:** 1 World Resources Institute, Washington, DC, United States of America; 2 Faculty of Technology, Policy and Management, Delft University of Technology, Delft, Netherlands; 3 Raise Green, Inc., Somerville, Massachusetts, United States of America; 4 Graduate School of Business, Stanford University, Stanford, California, United States of America; 5 School of Public Policy, University of North Carolina-Chapel Hill, Chapel Hill, North Carolina, United States of America; Texas A&M University, UNITED STATES

## Abstract

Research on the economic burden of air pollution has focused primarily on its macroeconomic impact. However, as some studies have found that air pollution can lead to avoidance behavior–for example, reducing the time spent outdoors–we hypothesize that it can also influence consumer spending activity. We combine high frequency data on ozone and fine particulate pollution with daily consumer spending in brick-and-mortar retail in 129 postal codes in Spain during 2014 to estimate the association between the two. Using a linear fixed effects model, we find that a 1-standard deviation increase in ozone concentration (20.97 μg/m^3^) is associated with 3.9 percent decrease in consumer spending (95% CI: -0.066, -0.012; p<0.01). The association of fine particulate matter with consumer spending is, however, not statistically significant (β: 0.005; 95% CI: -0.009, 0.018; p>0.10). Further, we do not observe a sufficiently strong bounce-back in consumer spending in the day–or even the week–following higher ozone concentration. Also, we find that the relationship between ozone concentration and consumer spending is heterogeneous, with those aged below 25 and those aged 45 or above exhibiting stronger negative association. This research informs policymakers about a plausibly unaccounted cost of ambient air pollution, even at concentrations lower than the WHO air quality guideline for short-term exposure.

## 1. Introduction

According to the latest State of the Global Air Report, over 90 percent of the global population lives in areas with unsafe air [1]. Short-term and long-term exposure to air pollution have been known to cause morbidity and mortality; evidence on the adverse effect of air pollution on various disabilities and diseases–such as acute respiratory illness, cardiovascular disease, and impaired cognitive performance [2–4]–has been growing [5]. In 2017 alone, air pollution was estimated to account for five million premature deaths, making it the leading environmental killer worldwide (1). As per one study, the economic burden of air pollution in 2013 –due to loss in labor and premature mortality–was estimated to be over USD 5 trillion [6]. Without concerted action, these losses are likely to persist: the Organization for Economic Co-operation and Development (OECD) has forecasted that air pollution might decrease the global gross domestic product (GDP) by one percent even in 2060, due to labor and productivity loss, crop damage, and increase in healthcare expenditure [7].

The microeconomic impact of air pollution, however, is less well understood. Some studies find that–for example, due to its adverse effect on visibility–air pollution is associated with annoyance, stress, and depression [8–10]. As a result, air pollution might result in changes in individual behavior, such as substantial reduction in the time spent outdoors and decrease in preference for tourist activities [9, 11]. Illustratively, in their study on air quality in Los Angeles, Breshanan et al. [12] find that nearly two-thirds of the respondents reported limiting or restricting outdoor leisure activities on days with poor air quality. This phenomenon has been termed as avoidance behavior [13, 14]. When individuals ‘avoid’ air pollution exposure, their pattern of consumer spending might be affected [15], as observed in the case of non-healthcare spending in Chinese cities due to short-term variation in PM_2.5_ [16]. Conversely, prior research has also reported an increase in online spending–moderated by age group, with younger consumers exhibiting a stronger preference–due to avoidance behavior during periods of elevated air pollution [17]. Such adjustment can occur voluntarily or without conscious awareness, even at a low level of pollution [8, 18].

This evidence leads us to suspect that people adjust their economic behavior to mitigate the risk of air pollution exposure, thereby affecting retail consumer spending activity. We hypothesize that, after controlling for factors that influence consumer spending–such as age–air pollution is negatively associated with retail consumer spending. In this study, we analyze this relationship in the case of Spain. Although Spain has been referred to as “the most polluted country in Europe” based on exceedance of the ozone (O_3_) air quality threshold [19], it is less polluted than most low- and middle-income countries [20, 21]. Therefore, the range of air pollution captured in our sample is likely to overlap with several countries around the world for at least some part of the year.

While several pollutants contribute to ambient air pollution, we focus specifically on O_3_ and fine particulate matter (PM_2.5_) as those pollutants contribute substantially to smog, can persist in the atmosphere for days, and are known to have a particularly adverse effect on human health [22, 23]. The primary source of PM_2.5_ pollution in Spain is fossil fuel combustion for electricity generation (56 percent) and transportation (34 percent), while O_3_ is formed secondarily from reactions of precursor gases–such as nitrogen oxides, carbon monoxide, methane, and non-methane volatile organic compounds emitted from fossil fuel combustion–rather than emitted directly [24]. Further, household consumption is a key constituent of overall economic activity of any country and has consistently accounted for over 50 percent of the GDP in Spain [25]. Due to data availability, we concentrate on credit and debit card spending only in brick-and-mortar retail.

By combining spatially-explicit, high-resolution, daily data on air quality with information on daily consumer spending for 129 postal codes in Spain, we evaluate the change in credit and debit card spending as a function of ambient O_3_ and PM_2.5_, after controlling for other factors that might affect consumer spending, such as weather, and incorporating postal code fixed effect, day fixed effect, and monthly trend by postal code. Our study design cannot rule out endogeneity between ambient air pollution and consumer spending. For example, unobserved characteristics such as economic shocks or short-lived political events could affect both economic activity and air pollution, or consumers could simply be shifting from retail spending to online spending [16]. Yet, we provide evidence on their relationship based on spatially and temporally granular data with a large set of control variables. We contribute to the literature by showing that O_3_ exposure and consumer spending are related, even at a moderate level of air pollution. Further, the magnitude of their association might even be comparable to the economic burden of air pollution due to lost labor, crop damage, and healthcare expenditure. Therefore, this relationship merits more attention than it has received thus far. Through this study, we provide support to a growing body of literature on avoidance behavior and the economic impact of air pollution.

## 2. Methods

### 2.1 Data collection and preparation

Our analysis is based on daily debit and credit card data of retail spending during 2014 in 129 postal codes in Spain, provided by the Banco Bilbao Vizcaya Argentaria (BBVA). These postal codes are in the inner province of Madrid, 11 provinces located along the Mediterranean coast (including Barcelona and Valencia), and the Balearic Islands. The data are available as aggregate debit and credit card spending by BBVA customers in Spanish retail stores by age group (below 25, 25–34, 35–44, 45–54, 55–64, 65 and above) per postal code at daily frequency.

Our data are unbalanced (i.e., meaning there are an unequal number of observations per postal code) as data for some postal codes are missing for several days, especially during the latter part of the year. Yet, the dataset contains over 60,000 observations by age group per postal code and over 10,000 observations aggregated at the postal code level. As data to compare the profile of an ‘average’ BBVA customer with that of an ‘average’ consumer of Spain is unavailable, we cannot comment on the representativeness of the data. Regardless, the share of BBVA in the retail market in Spain is approximately 12 percent [26], and an analysis of this spatially and temporally granular—but otherwise proprietary—data can shed light on the plausible impact of air pollution on microeconomic activity within the sample.

We combine the data on consumer spending with data on air pollution, specifically O_3_ and PM_2.5_, which are among the most harmful pollutants for human health and contributors to smog [22]. First, we obtain data on the location of all O_3_ and PM_2.5_ monitoring stations in Spain from the European Environment Agency [27]. Next, we obtain high-frequency data on pollutant concentrations for all O_3_ and PM_2.5_ monitoring stations. While most observations correspond to the daily averages of the O_3_ or PM_2.5_ concentrations measured at the monitoring station (Averaging Time = ‘day’), some correspond to the hourly averages measured on a given day (Averaging Time = ‘hour’). In case of the latter, we calculate the daily concentrations per monitoring station by averaging all hourly readings available for the pollutant for that day. Subsequently, we match each postal code to the nearest O_3_ and PM_2.5_ monitoring stations, individually, based on the centroid of the postal code and the location of the monitoring station. The daily O_3_ and PM_2.5_ exposures per postal code are assigned as the pollutant concentrations at the nearest monitoring stations. Where the nearest monitoring station has not recorded the pollutant concentrations on a given date, the value is left as missing rather than being assigned from a different monitoring station, which might increase measurement error. In such a case, the corresponding observation on spending is discarded from subsequent analysis. In our final dataset, the distance of an O_3_ monitoring station from the postal code is in the range 0.27–31.56 km (mean: 1.96; SD: 2.91), while that of a PM_2.5_ monitoring station is in the range 0.31–128.78 km (mean: 6.36; SD: 13.14). While we do not exclude observations from monitoring stations that are located beyond a certain threshold, we confirm that this does not affect our main result (see *section 3*.*4*).

In addition, we include data on daily weather in our analysis. We use hourly, spatial data for 2-meter temperature (°C), surface pressure (Pa), total precipitation (m), and 2-m dewpoint temperature (°C) during 2014 from the ERA-5 Land dataset of the European Monitoring Centre for Medium-Range Weather Forecasts (ECMWF). The ERA5-Land is a publicly available climate reanalysis dataset, gridded at a resolution of approximately 9 km by 9 km [28]. We match this dataset with a geospatial map containing boundaries of all the postal codes in Spain using the following procedure. First, we average hourly readings to obtain the daily mean temperature and mean pressure for each grid cell. Second, we sum hourly readings to obtain the daily total rainfall for each grid cell. Third, we calculate the daily mean for all grid cells that spatially overlapped a postal code.

**Fig 1:**
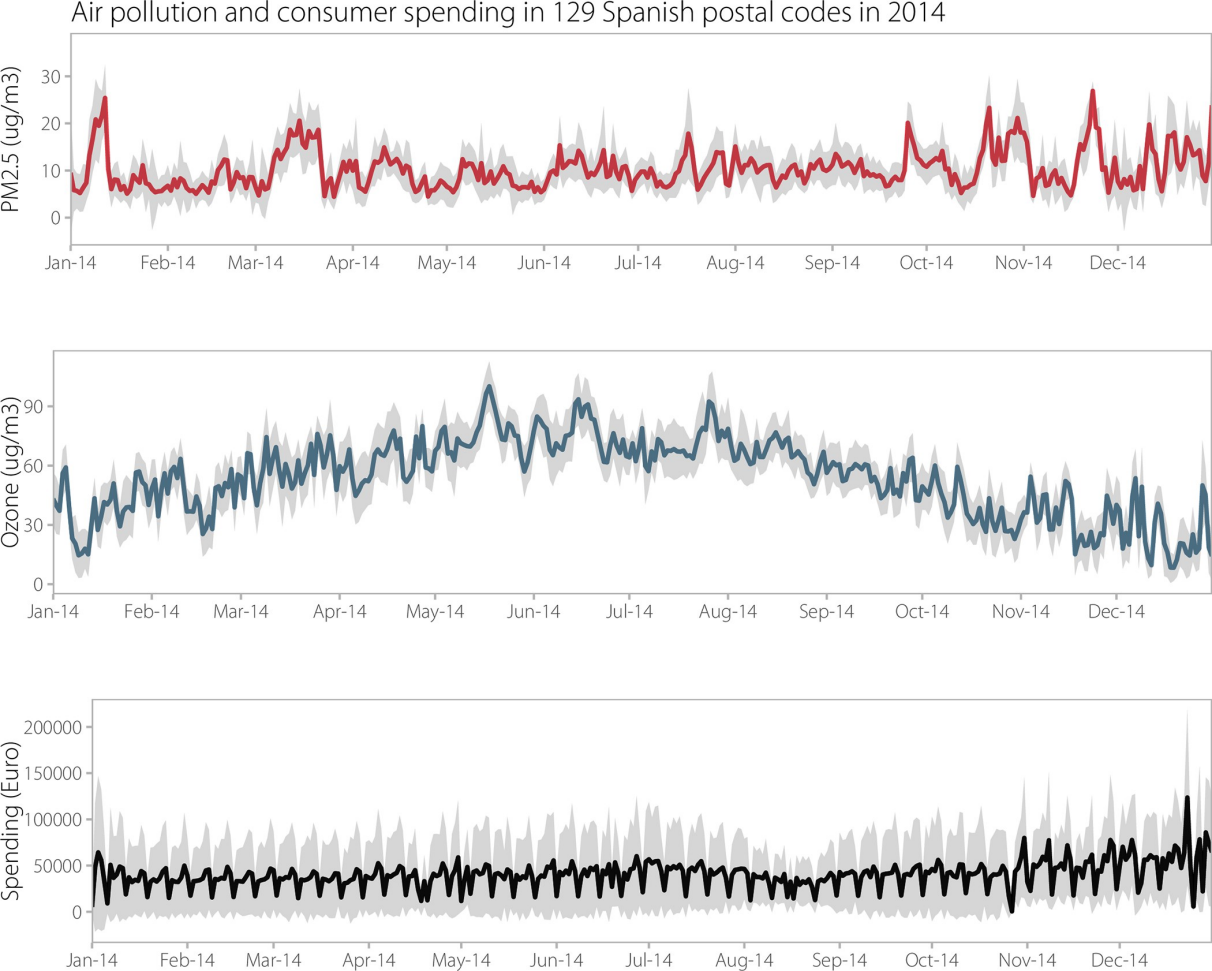
Average O_3_ and PM_2.5_ pollutant concentration and consumer spending by postal code (n = 129) in Spain in 2014. The grey shaded area represents ±1 standard deviation from the mean values.

As a result, our final dataset consists of consumer spending, O_3_ and PM_2.5_ concentrations, and weather per postal code per day. Table 1 presents the summary statistics for the key variables of this study. While the average consumer spending per postal code per day in our sample was EUR 38,038 (see also S1 Table in S1 File), it varied substantially by day of the week (Fig 1). The average consumer spending per postal code was especially low on Sundays (EUR 17,715) and much higher on Fridays and Saturdays (EUR 47,405 and EUR 44,389, respectively). Also, average consumer spending per postal code increased over the course of the year, from EUR 35,260 in the first quarter to EUR 38,503 in the next quarter, and EUR 45,182 during October-December. In our dataset, the mean daily consumer spending was the lowest for postal codes in the municipalities of Murcia and Granada (approximately EUR 1960 and 11,032, respectively, for the postal codes with the lowest mean) and the highest for the municipalities of Barcelona and Madrid (~ EUR 47,600 and EUR 52,782, respectively, for the postal codes with the highest mean).

**Table 1:** Descriptive statistics for the study area.

	N	Min	Max	Mean	S.D.
Spending (€)	10948	195.57	440564.09	38037.91	42732.38
O_3_ (μg/m^3^)	10948	1.00	128.96	57.27	20.97
PM_2.5_ (μg/m^3^)	10948	0.00	66.00	9.97	5.83
Temperature (°C)	10948	-0.02	30.18	16.04	6.71
Rainfall (m)	10948	0.00	0.07	0.00	0.00
Pressure (Pa)	10948	88714.97	102276.41	96363.67	3343.27
Dewpoint temperature (°C)	10948	-11.60	23.87	7.79	5.69

The mean concentrations for O_3_ and PM_2.5_ were 57.27 μg/m^3^ and 9.97 μg/m^3^, respectively, below the WHO air quality guidelines of 100 μg/m^3^ and 25 μg/m^3^ for short-term exposures in each case (Table 1). Daily O_3_ pollution tended to be slightly higher on weekends (60.35 μg/m^3^) as opposed to weekdays (56.05 μg/m^3^), while daily PM_2.5_ pollution tended to be slightly higher on weekdays (10.14 μg/m^3^) versus weekends (9.52 μg/m^3^). Also, average daily O_3_ pollution was the highest during the second quarter and lowest during the fourth quarter (70.73 μg/m^3^ versus 36.66 μg/m^3^), whereas average daily PM_2.5_ pollution was the lowest during the second quarter and highest during the fourth quarter (9.28 μg/m^3^ versus 11.65 μg/m^3^). The descriptive statistics by quarter of the year for the are shown in Table 2.

**Table 2:** Descriptive statistics by quarter of the year.

Variable\Quarter	1		2		3		4	
	Mean	S.D.	Mean	S.D.	Mean	S.D.	Mean	S.D.
Spending (€)	35259.63	42924.83	38503.41	43751.71	38369.06	39056.44	45182.36	46645.44
O_3_ (μg/m^3^)	47.59	18.71	70.73	17.24	64.07	15.23	36.66	16.81
PM_2.5_ (μg/m^3^)	9.78	6.80	9.28	4.84	10.30	4.82	11.65	6.61
Temperature (°C)	9.34	3.40	18.04	3.77	23.56	3.04	14.93	5.37
Rainfall (m)	0.00	0.00	0.00	0.00	0.00	0.00	0.00	0.01
Pressure (Pa)	96507.12	3412.19	96489.88	3348.33	96211.19	3273.93	95910.17	3208.30
Dewpoint (°C)	3.52	3.16	8.33	4.50	12.33	5.66	9.69	5.17

**Fig 2:**
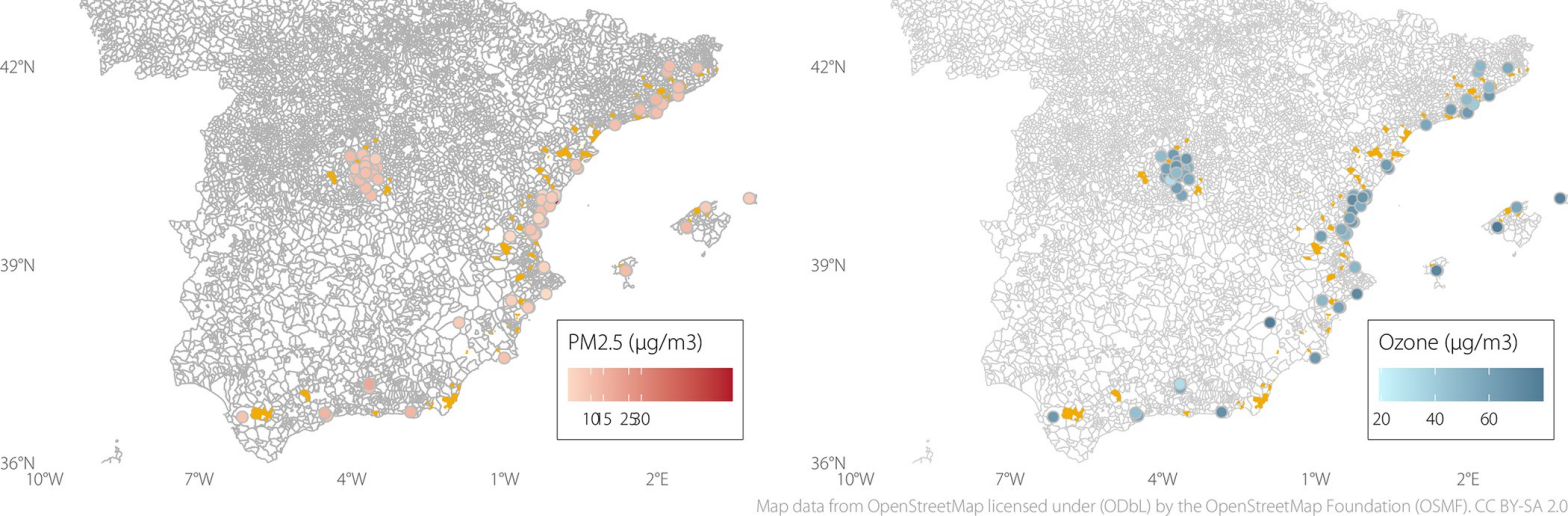
Annual mean O_3_ and PM_2.5_ concentration in 2014 for postal codes in the study. Areas shaded in yellow correspond to the area of the postal codes. The markers indicate the location of pollution monitoring stations from the European Environment Agency. Maps were generated from OpenStreetMap tiles (© OpenStreetMap contributors: https://www.openstreetmap.org/copyright).

Fig 2 illustrates the spatial variation in air pollution across the study areas. On average, postal codes in Balears and Almeria municipalities had the highest average daily O_3_ pollution (mean: 76.14 and 69.05 μg/m^3^, respectively), while those in Granada and Barcelona had the lowest (mean: 52.47 and 53.86 μg/m^3^, respectively). In the case of PM_2.5_, postal codes in the municipalities of Almeria and Granada had the highest daily average PM_2.5_ concentration (13.99 and 12.94 μg/m^3^ respectively), while those in Alicante and Castellon had the lowest (7.82 and 8.08 μg/m^3^ respectively).

### 2.2 Main specification

A key concern while estimating the relationship between consumer spending and air pollution is the endogeneity of the latter. Several unobserved socioeconomic characteristics might influence both air pollution as well as consumer spending. To ascribe causality, a quasi-experimental design relying on exogenous variation in air pollution or an instrument variable (IV) approach is necessary. As we do not have spatially and temporally granular data for such a design, our estimated coefficients cannot be interpreted as the effect of air pollution on consumer spending. However, we develop a fixed effects model to estimate the association of air pollution, namely O_3_ and PM_2.5_, with consumer spending while controlling for several observed characteristics.

For the main result, we estimate the impact on consumer spending *S* in postal code of the spending location *z* on date *t* using the following specification:

$$S_{t,z}=\alpha +\beta {O3}_{t,z}+\gamma {PM2.5}_{t,z}+\delta_{1}{Temperature}_{t,z}+\delta_{2}{Rainfall}_{t,z}+\delta_{3}{Pressure}_{t,z}+\delta_{4}{Dewpoint}_{t,z}+\zeta_{z}+\eta_{t}+\theta_{z}*{Month}_{t}+\varepsilon_{t,z}$$ (Eq 1)

In this equation, *S*_*t*,*z*_ is the natural logged (ln) credit and debit card spending (in Euro) in postal (zip) code of the spending location *z* on date *t*, *O*3_*t*,*z*_ is the concentration of O_3_ (in μg/m^3^), and *PM*2.5_*t*,*z*_ is the concentration of PM_2.5_ (in μg/m^3^). We control for daily weather characteristics that might influence consumer behavior and, hence, spending [29–31]: *Temperature*_*t*,*z*_ is the 24-hour mean temperature (in degree Celsius), *Rainfall*_*t*,*z*_ is the 24-hour total rainfall (in meter), *Pressure*_*t*,*z*_ is the 24-hour mean pressure (in Pascal), and *Dewpoint*_*t*,*z*_ is the dewpoint temperature (in degree Celsius).

We include fixed effects in our model: *ζ*_*z*_ is the postal code fixed effect to control for unobserved, time-invariant characteristics that are common within a postal code for the duration of our study (for example, the ability to spend); and *η*_*t*_ is the date fixed effect to control for exogenous characteristics that influence spending in all postal code on a given day (for example, holidays). Also, *θ*_*z*_ is a linear monthly trend in consumer spending specific to each postal code (illustratively, gradual changes in the number and type of retail stores in a specific postal code during the year).

Finally, *ε*_*t*,*z*_ is the error term. We cluster standard errors by postal code *z* and the date *t*. Further, all independent variables and control variables are normalized using the scale function in R, which centers data with mean (μ) = 0 and standard deviation (**σ**) = 1. See robustness checks for other specifications considered in the analysis (Section 3.4).

### 2.3 Temporal displacement

Air pollution ma affect the timing of the spending, i.e., displace it temporally rather than reduce it permanently. For example, consumers may avoid going out on a day with high air pollution, but instead go out (and spend in a retail store) on a subsequent low air pollution day. In such a scenario, where a high pollution day is followed by a low pollution one–after controlling for present-day air pollution–we would expect a positive association of one-day prior air pollution with present-day consumer spending. Therefore, we examine whether consumer spending exhibited temporal displacement using the following fixed effects specification:

$$S_{t,z}=\alpha +\beta_{1}{O3}_{t,z}+\beta_{2}{O3}_{t-1,z}+\gamma_{1}{PM2.5}_{t,z}+\gamma_{2}{PM2.5}_{t-1,z}+\delta_{1}{Temperature}_{t,z}+\delta_{2}{Temperature}_{t-1,z}+\delta_{3}{Rainfall}_{t,z}+\delta_{4}{Rainfall}_{t-1,z}+\delta_{5}{Pressure}_{t,z}+\delta_{6}{Pressure}_{t-1,z}+\delta_{7}{Dewpoint}_{t,z}+\delta_{8}{Dewpoint}_{t-1,z}+\zeta_{z}+\eta_{t}+\theta_{z}*{Month}_{t}+\varepsilon_{t,z}$$ (Eq 2)

In this equation, we include the present-day and the one-day prior (i.e., the first lag) values of each pollutant. In addition, we include the lags of temperature, rainfall, pressure, and dewpoint temperature as well. It is plausible that temporal displacement might occur over a longer time period. For example, some people might delay some types of expenditure by a week or even longer. To test for this possibility, we modify Eq (2) to include values of air pollution (*O*_*3*_ and *PM*_*2*.*5*_) and weather (*Temperature*, *Rainfall*, *Pressure*, and *Dewpoint*) from the present day until their sixth lag.


$$S_{t,z}=\alpha +\sum_{k=0}^{6}\beta_{t-k}{O3}_{t-k,z}+\sum_{k=0}^{6}\gamma_{t-k}{PM2.5}_{t-k,z}+\sum_{k=0}^{6}\delta_{1,t-k}{Temperature}_{t-k,z}+\sum_{k=0}^{6}\delta_{2,t-k}{Rainfall}_{t-k,z}+\sum_{k=0}^{6}\delta_{3,t-k}{Pressure}_{t-k,z}+\sum_{k=0}^{6}\delta_{4,t-k}{Dewpoint}_{t-k,z}+\zeta_{z}+\eta_{t}+\theta_{z}*{Month}_{t}+\varepsilon_{t,z}$$ (Eq 3)

It is also plausible that the coefficients for O_3_ or PM_2.5_ estimated based on Eq 3 might be statistically significant jointly rather than individually. Therefore, we conduct a joint nullity test for O_3_ (i.e., ***β*** = 0) and for PM_2.5_ (i.e., ***γ*** = 0) using the Wald test statistic. Further, we estimate the effect of week-long exposure to O_3_ on consumer spending on a given day by estimating the coefficient and standard error of the linear combination of regression coefficients (i.e., $\sum_{k=0}^{6}\beta_{t-k}$).

### 2.4 Heterogeneity in response by age group

Also, the association of air pollution with consumer spending could differ by age group. For example, an increase in air pollution might have a stronger relationship with spending by the elderly (say, age group 65 and above) than that by younger people (say, the age group 25–34). We interact the air pollution variables with the age group category to examine whether association of air pollution with consumer spending varies by age group. Specifically, we use the following interaction effects specification:

$$S_{a,t,z}=\alpha +\beta {O3}_{t,z}*a+\gamma {PM2.5}_{t,z}*a+\delta_{1}{Temperature}_{t,z}+\delta_{2}{Rainfall}_{t,z}+\delta_{3}{Pressure}_{t,z}+\delta_{4}{Dewpoint}_{t,z}+\kappa *a+\zeta_{z}+\eta_{t}+\theta_{z}*{Month}_{t}+\varepsilon_{t,z}$$ (Eq 4)

In this equation, *S*_*a*,*t*,*z*_ is the natural logged (ln) credit and debit card spending (in Euro) by age group *a* in postal code of the spending location *z* on date *t*, ***κ*** is the vector representing age group effect while *ζ*_*z*_ is the postal code fixed effect. The age group specific associations of air pollution and consumer spending for O_3_ and PM_2.5_ are given by ***β*** and ***γ***, respectively. In case of heterogeneity in response by age group, we expect the coefficients in ***β*** and/or ***γ*** to differ in their size, sign, and/or statistical significance.

### 2.5 Software

Data on air pollution and weather are collected using Python version 3.7 [32]. Analyses are performed in R (version 3.52) [33] using the *scales* package [34] for normalization, the *stats* package [35] ‘feols’ function for fitting linear fixed effects models, the fixest package [36] ‘wald’ function to test the joint nullity of a set of coefficients, and the multcomp package [37] ‘glht’ function for testing general linear hypotheses. Figures are made using the *ggplot* package [38] and the ‘coefplot’ function in the fixest package [36].

## 3. Results

### 3.1 Relationship between air pollution and consumer spending

A correlational analysis between air pollution and total consumer spending presents a mixed picture. During the fourth quarter of the year, when average spending is highest, days that were in the top quintile of O_3_ pollution (S2 Fig in S1 File, shaded in red) generally had lower spending on average than days that were in the bottom quintile of O_3_ pollution (S2 Fig in S1 File, shaded in blue). However, this pattern was even more mixed for other quarters when O_3_ pollution tended to be higher. In the case of PM_2.5_, on the other hand, the correlation with mean consumer spending appeared to vary based on day of the week and no consistent pattern was present in any quarter of the year (S3 Fig in S1 File).

The main results of our model (Eq 1) show that a higher level of O_3_ pollution is associated with lower consumer spending (Table 3). In the complete specification (Table 3: column 4), we find that an increase of 1 standard deviation in O_3_ pollution (20.97 μg/m^3^) is associated with a 3.9 percent decrease in consumer spending (95% CI: -0.066, -0.012; p<0.01). The relationship between PM_2.5_ pollution and consumer spending, on the other hand, is not statistically significant. An increase of 1 standard deviation in PM_2.5_ pollution (5.83 μg/m^3^) is associated with a 0.5 percent increase in consumer spending (95% CI: -0.009, 0.018; p>0.10). Among the control variables for weather, temperature is negatively associated with consumer spending (β: -0.117; 95% CI: -0.213, -0.020; p<0.05) while dewpoint temperature is positively associated with consumer spending (β: -0.054; 95% CI: 0.001, 0.108; p<0.05).

**Table 3:** The regression of consumer spending on air pollution.

	(1)	(2)	(3)	(4)
O_3_	-0.056***	-	-0.056***	-0.039**
	[-0.088, -0.024]		[-0.088, -0.024]	[-0.066, -0.012]
PM_2.5_		0.004	0.000	0.005
		[-0.010, 0.018]	[-0.013, 0.013]	[-0.009, 0.018]
Temperature	-	-	-	-0.117*
				[-0.213, -0.020]
Rain	-	-	-	0.012
				[-0.004, 0.027]
Pressure	-	-	-	-0.090
				[-0.457, 0.277]
Dewpoint temperature	-	-	-	0.054*
				[0.001, 0.108]
N	10948	10948	10948	10948
R^2^	0.919	0.919	0.919	0.921
R^2^ Adjusted	0.915	0.915	0.915	0.917

Notes: The unit of analysis is postal code with daily frequency. The dependent variable is the log of total spending. The independent variables and control variables have been normalized. The regressions include age group by postal code fixed effect, date fixed effect, and monthly trend by postal code. The standard errors in brackets are clustered by postal code and date. + p < 0.1

* p < 0.05

** p < 0.01

*** p < 0.001.

### 3.2 Temporal displacement effect

If O_3_ exposure were associated with temporal displacement by a day (rather than a reduction) in consumer spending, we would expect the first lag of O_3_ to have a positive, statistically significant association with consumer spending, after controlling for the present-day concentration of O_3_ (i.e., in Eq 2). In the regression based on Eq 2 (S2 Table, column 1 in S1 File), we find that the present-day concentration of O_3_ has a consistent and statistically significant association with consumer spending (β: -0.034; 95% CI: -0.067, -0.001; p<0.05), even after controlling for its first lag. In contrast, relationship between the first lag of O_3_ and consumer spending is not statistically significant (β: 0.008; 95% CI: -0.030, 0.047; p>0.10). Thus, ceteris paribus, high O_3_ concentration on the previous day does not lead to an increase in consumer spending on the present day.

**Fig 3:**
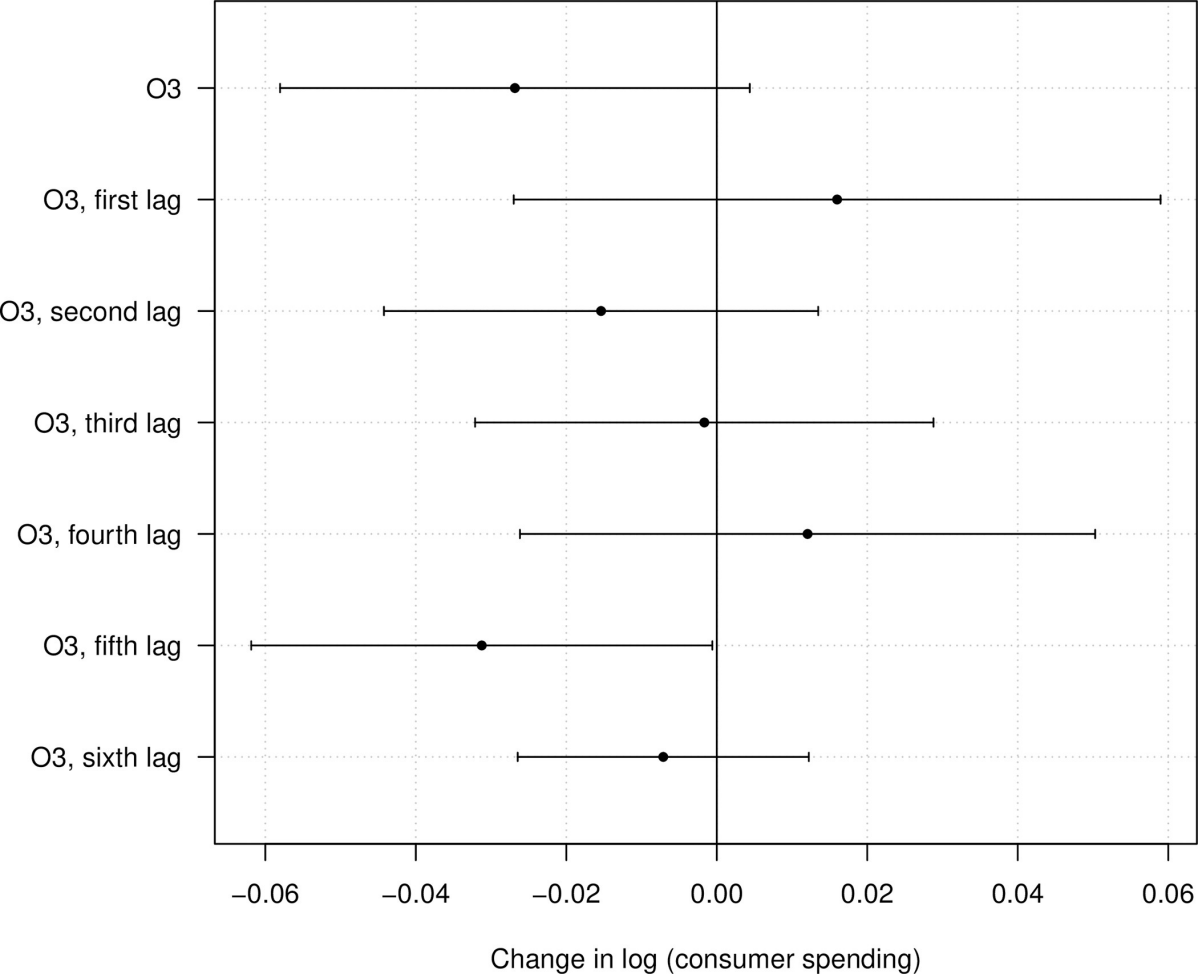
The association of O_3_ concentration and consumer spending. The plot is based on the regression in S2 Table, column 6 in S1 File.

Even after controlling for lagged O_3_ over a longer period, we do not find evidence for temporal displacement of consumer spending (S2 Table in S1 File). Fig 3 shows the estimated beta coefficients of present-day O_3_ as well as its lagged concentration for six days (see also S2 Table, column 6 in S1 File). Here, we see that a 1 standard deviation increase in the present-day concentration of O_3_ is marginally associated with a 2.7 percent reduction in consumer spending (95% CI: -0.059, 0.005; p<0.10). Further, the fifth lag of O_3_ also has a marginally strong association with consumer spending (β: -0.031; 95% CI: -0.063, -0.000; p<0.10). In addition, no lag of O_3_ has a statistically significant, positive association with consumer spending. The joint hypothesis test for O_3_ concentrations in this specification has a Wald statistic of 1.43 (p>0.10). Based on a general linear hypothesis test, however, a 1 standard deviation increase in O_3_ pollution for an entire week is associated with a 5.4 percent reduction in consumer spending (SE: -0.023; p<0.05). As per this estimate, the association of a 1 standard deviation increase in the O_3_ concentration over an entire week is much less than the cumulative daily association, indicating complex temporal dynamics between lagged O_3_ and consumer spending.

### 3.3 Heterogeneity by age group

Finally, the results of an analysis based on age group reveal some heterogeneities in the response (Table 4, column 2). For the age group less than 25, an increase of 1 standard deviation in O_3_ pollution is associated with a 4.9 percent decrease in consumer spending (95% CI: -0.090, -0.009; p<0.05). The association of O_3_ concentration with consumer spending for those who are 45 and above (i.e., age groups 45–54, 55–64, and > = 65) is also statistically significant. In contrast, O_3_ pollution has a smaller (albeit still negative) association with consumer spending for those in the age groups 25–34 and 35–44. This indicates that O_3_ pollution has a more negative association with consumer spending for those aged < 25 and for those aged > = 45. Surprisingly, PM_2.5_ pollution has a statistically significant, positive association with consumer spending for those aged < 25, although not for any other age group. As mentioned below (see section 3.4), this might indicate some reverse causality between consumer spending and PM_2.5_ concentration and would require assessment based on more granular data.

**Table 4:** The interaction effect of O_3_ with age.

	(1)	(2)
O_3_	-0.043**	-
	[-0.072, -0.014]	
PM_2.5_	0.005	-
	[-0.008, 0.019]	
O_3_ * Age group, < 25		-0.049*
		[-0.090, -0.009]
O_3_ * Age group, 25–34	-	-0.008
		[-0.043, 0.028]
O_3_ * Age group, 35–44	-	-0.004
		[-0.035, 0.027]
O_3_ * Age group, 45–54	-	-0.044**
		[-0.077, -0.012]
O_3_ * Age group, 55–64	-	-0.067***
		[-0.102, -0.033]
O_3_ * Age group, > = 65	-	-0.088***
		[-0.135, -0.041]
PM_2.5_ * Age group, < 25	-	0.051*
		[0.012, 0.090]
PM_2.5_ * Age group, 25–34	-	0.016
		[-0.017, 0.050]
PM_2.5_ * Age group, 35–44	-	0.006
		[-0.022, 0.033]
PM_2.5_ * Age group, 45–54	-	-0.004
		[-0.025, 0.017]
PM_2.5_ * Age group, 55–64	-	-0.017
		[-0.042, 0.007]
PM_2.5_ * Age group, > = 65	-	-0.021
		[-0.070, 0.029]
Temperature	-0.122*	-0.122*
	[-0.219, -0.025]	[-0.218, -0.025]
Rain	0.013+	0.013+
	[-0.002, 0.027]	[-0.002, 0.027]
Pressure	-0.103	-0.101
	[-0.472, 0.266]	[-0.470, 0.269]
Dewpoint temperature	0.052+	0.052+
	[-0.003, 0.106]	[-0.003, 0.106]
Age group, 25–34	2.032***	2.033***
	[1.905, 2.159]	[1.908, 2.158]
Age group, 35–44	2.493***	2.494***
	[2.322, 2.665]	[2.326, 2.663]
Age group, 45–54	2.258***	2.258***
	[2.111, 2.405]	[2.114, 2.403]
Age group, 55–64	1.799***	1.800***
	[1.644, 1.954]	[1.648, 1.951]
Age group, > = 65	1.312***	1.313***
	[1.085, 1.539]	[1.088, 1.538]
N	63881	63881
R^2^	0.873	0.874
R^2^ Adj.	0.872	0.873

Notes: The unit of analysis is age group by postal code with daily frequency. The dependent variable is the log of total spending. The independent variables and control variables have been normalized. The regression includes postal code fixed effect, date fixed effect, and monthly trend by postal code. The standard errors in brackets are clustered by postal code and date. + p < 0.1

* p < 0.05

** p < 0.01

*** p < 0.001.

### 3.4 Robustness checks

We examine the sensitivity and robustness of our findings in several ways. First, we analyze variations of our main model (Eq 1) by: i) excluding weather; ii) excluding PM_2.5_ but retaining O_3_; and, iii) excluding O_3_ but retaining PM_2.5_ (Table 2, columns 1–3). The results show that our estimates are robust for both O_3_ and PM_2.5_. Second, we also examine the association of consumer spending with one-day prior air pollution, i.e., its first lag, instead of the present-day concentration (S3 Table in S1 File). This is different from the temporal displacement conducted above, which included both the present-day concentration as well as the first lag. As one might expect, we find that the first lag of O_3_ has a weaker association than its present-day concentration with consumer spending. Surprisingly, in contrast, the first lag of PM_2.5_ has a more negative, statistically significant association than its present-day concentration with consumer spending. This might indicate a delayed effect in the case of PM_2.5_ or reverse causality between the present-day concentration and consumer spending, whereby activities that facilitate consumer spending also contribute to an increase in PM_2.5_ (for example, motorized travel). Third, we show that the findings are reasonably robust to the functional specification of air pollutant by running regressions with the log form, the quadratic form, and a cubic spline of O_3_ pollution (S4 Table in S1 File). While the estimates from the spline specification suggest nonlinearity in the relationship between consumer spending and O_3_ pollution, their confidence interval is wide. Therefore, we retain a linear specification for the main model for ease of interpretation and for reducing the likelihood of overfitting. Fourth, we confirm the robustness of the estimates for O_3_ to variation in the type of fixed effect (S5 Table in S1 File). Fifth, to check whether our findings are affected by measurement bias introduced due to the inclusion of postal codes that are far from the pollution monitoring stations, we conduct subgroup analysis based on distance to monitoring station (S6 Table in S1 File). For this analysis, we segregate our dataset into observations with both O_3_ and PM_2.5_ monitoring stations at a distance of 10 km or less (‘Nearby station’) and observations with either or both monitoring stations at a distance more than 10 km (‘Distant station’). We find that the size of the O_3_ coefficient is comparable for both subgroups, although the estimate is statistically significant for the subgroup with ‘nearby station’, but not for the subgroup with ‘distant station’. As about 80 percent of the observations in our dataset are in the ‘Nearby station’ category, this might be due to low statistical power. Sixth, to test whether the relationship between air pollution and consumer spending is sensitive to the typical air pollution level in the region (i.e., whether spending in a less polluted postal code is less responsive to an increase in air pollution as people might not perceive air pollution as a problem there), we conduct subgroup analysis for observations from postal codes whose average air pollution in our dataset is less than 50 μg/m^3^ for O_3_ and 10 μg/m^3^ for PM_2.5_ (S7 Table in S1 File). While the estimated coefficient for O_3_ for the subgroup with low O_3_ pollution is approximately comparable in magnitude to that of our main specification, its confidence interval is rather wide. This might suggest that the relationship between air pollution and consumer spending varies based on the typical level of air pollution, but could also be due to low statistical power (as the number of observations in our dataset for this analysis is only approximately 2,500).

## 4. Discussion and conclusion

Our study provides preliminary evidence on the relationship between ambient air pollution and consumer spending in the form of daily debit and credit card transactions in brick-and-mortar retail in 129 postal codes in Spain during 2014. We find that: (i) an increase of 1 standard deviation in O_3_ pollution (20.97 μg/m^3^) is associated with a 3.9 percent decrease in consumer spending; (ii) the present-day concentration of O_3_ has a strong association with consumer spending, even after controlling for the first lag of O_3_ concentration. Meanwhile, after controlling for the present-day O_3_ concentration, the relationship between the first lag of O_3_ and consumer spending is not statistically significant, indicating that consumer spending is likely to be reduced–and not only temporally displaced [16]–at least in the short-term; and, (iii) the association of O_3_ concentration with consumer spending varies by age group, with spending among those below 25 and those above 45 exhibiting a stronger relationship than among those between 25–44. Thus, our findings suggest that O_3_ pollution–even at a moderate level–might reduce retail consumer spending, thereby inflicting a previously unaccounted economic cost.

Although research on the relationship between air pollution and consumer spending is limited, our findings are broadly in line with previous studies. For example, Barwick et al. [16] analyze the influence of PM_2.5_ on healthcare and non-healthcare spending in China by leveraging spatial spillover of PM_2.5_ and using a flexible distributed lag model. They argue that PM_2.5_ concentration has a positive effect on healthcare spending but a negative effect on non-healthcare spending in the short-term. Possibly due to differences in the study design and the study setting–we do not disaggregate healthcare and non-healthcare spending, do not use the instrumental variables technique, and examine a setting with much lesser PM_2.5_ pollution–we do not find the association between PM_2.5_ and consumer spending to be statistically significant. However, we too find that air pollution (in our case, O_3_) has a negative association with consumer spending as a whole.

In another study, Qiu et al. [17] study the effect of air pollution on online purchase behavior in China using a structural equation model. They find that air pollution–measured using the Air Quality Index provided by local Environmental Protection Bureaus in China–is associated with an increase in online consumption as opposed to traditional ‘offline’ consumption (*seemingly* in brick-and-mortar retail). Further, the relationship between air pollution and online consumer spending is likely to vary based on age, education, and income. While we do not have data on online spending, our findings corroborate the potential relationship between air pollution and non-online retail spending. Further, we also observe heterogeneities in the relationship between air pollution and retail spending based on age group.

A growing body of literature suggests that averting or avoidance behavior could explain the relationship between air pollution and consumer spending. Research has shown that O_3_ contributes to photochemical smog and decreases visibility [39]. Further, people living in more polluted areas score higher on anxiety and depression [40] and lower on happiness [41]. These psychological, as well as other physical, responses to air pollution–can result in ‘averting’ or ‘avoidance’ behavior. Bresnahan et al. [12], for example, report that people sensitive to O_3_ pollution adjust daily activities–for example, by spending less time outdoors–on days with high O_3_ pollution. Neidell [14] finds that such behavior is exhibited more strongly when information about air pollution is more prevalent, for example through smog alerts. In addition, Barwick et al. [16] and Qiu et al. [17] also report that avoidance behavior could be responsible for reduction in non-healthcare spending and offline spending, respectively, in China.

Existing studies suggest that daily activities might be temporally substituted rather than foregone altogether. Zivin and Neidell [42], for example, observe that when smog alerts are issued on successive days, avoidance behavior is significantly diminished on the second day. In their study on China, Barwick et al. [16] find that the cumulative effect of elevated PM_2.5_ concentration on healthcare spending persists for up to three months and the cumulative effect on non-healthcare spending lasts for about a month. While Barwick et al. [16] utilize a more comprehensive dataset on air pollution and spending spanning 2013–15, our dataset does not permit a long temporal analysis. However, our analysis does indicate that successive days of higher O_3_ concentration reduce its association with consumer spending substantially, although not all of the loss in consumer spending is likely to be made up within a week.

We also find heterogeneity by age group in the response to O_3_ pollution. Specifically, O_3_ concentration exhibits a stronger association with consumer spending by those below 25 and those above 45 than those aged between 25–44. The virtual non-responsiveness of those aged 25–44 to the O_3_ concentration might indicate that: (i) they are relatively less susceptible to air pollution, especially at the moderate level observed in our study setting; or (ii) a higher share of their retail spending is on more immediate, necessary goods and services. In contrast, the young and the elderly could be more sensitive to air pollution and, hence, more likely to exhibit avoidance behavior. Further, they might be able to defer some purchases or receive assistance from those aged between 25–44 in case of high air pollution. Finally, those aged below 25 might also be more able or willing to switch to online spending, as observed by Qiu et al. [17].

While this study focused on the association between air pollution and consumer spending, the evidence that the ambient air pollution has a causal effect on human health is growing. If the relationship between ambient air pollution is causal, our findings suggest that–even below the WHO air quality guidelines–ambient air pollution could be one driver of sustained economic losses due to reduced microeconomic activity. In fact, with a majority of the global population living in places that do not meet the present WHO air quality guidelines itself [43], the adverse microeconomic impact of air pollution is likely to be substantial.

We complement existing research on the economic cost of air pollution. Previous work has estimated the cost of air pollution due to lost labor, additional healthcare expenditure, and premature mortality to be 1–4 percent of GDP [6, 7]. Conversely, Tschofen et al. [44] have suggested that air pollution mitigation in the United States during 2008–14 decreased air-pollution related economic damage–due to reduction in premature mortality–by over 20 percent. Vrontisi et al. [45] have argued that even in Europe the benefit of a clean air policy would outweigh its cost when its positive macroeconomic feedback is considered. In our sample, a reduction of about 21 μg/m^3^ in the mean daily O_3_ level is associated with an increase of 3.9 percent in credit card consumer spending. Given that household consumption accounted for over half of the Spanish GDP [25], and that credit and debit cards are a key mode of payment, our findings suggest that there is an immediate economic cost of even moderate air pollution due to decreased activity. It is, however, possible that retail spending is substituted by online expenditure. Qiu et al. (2020), for example, found that air pollution caused an increase in people’s annoyance and anxiety, resulting in online shopping behavior [17]. Alternatively, a reduction in household consumption might lead to an increase in investment and savings. Even so, the association of air pollution with microeconomic activity–whether its extent, mode, or timing–is likely to be nontrivial and could disproportionately impact brick-and-mortar retail in comparison to e-commerce businesses.

Our analysis has numerous limitations that should be considered while interpreting its findings. First, our sample was limited to customers of BBVA in 129 postal codes in Spain and might suffer from selection bias. Second, our panel was unbalanced and data for several postal codes were limited to fewer than 100 days. Third, our variables on air pollution exposure are subject to measurement error due to the uneven distribution of pollution monitoring stations across Spain. Fourth, despite a rich set of controls for weather and other unobserved characteristics, we cannot rule out sources of variation that might be correlated with air pollution as well as consumer spending (such as sporting events and traffic). Fifth, we are unable to rule out any spillover effect–for example, on the use of cash or on online spending–due to lack of data. Further, hourly data on consumer spending and air pollution could have provided additional insight into the timing of exposure most relevant for the outcome. Also, data on sector-wise spending would have facilitated assessment of microeconomic activities (and, consequently, industries) associated with changes in consumer spending due to air pollution. Additionally, a longer time-series would have allowed us to control for unobserved, time-variant characteristics and made the study more robust.

Limitations notwithstanding, our sample consisted of daily credit and debit card spending in brick-and-mortar retail for postal codes in several municipalities in Spain and accounted for transactions worth EUR 833 million in 2014. We find that a moderate increase in air pollution, even at a relatively low level of ambient air pollution, is associated with a decrease in consumer spending. Additionally, this ‘loss’ in consumer spending is likely not entirely made up within a day, or even a week. Finally, the response to air pollution is heterogenous and, as a result, its association with consumer spending varies by age group. Our findings suggest that air pollution could reduce consumer spending and, thus, entail a previously unaccounted economic cost. In the absence of an appropriate policy response, its effects on the environment and human wellbeing are expected to worsen with climate change [46–48], already evidenced by increasing air pollution due to climate-induced wildfires that have become more common in recent years (Di Virgilio et al., 2019) [49] as well as increased temperatures overall leading to higher ozone and smog formation (Meleux et al., 2007) [50]. Policies that clean the air–by tackling local as well as transboundary pollution–will not only alleviate its macroeconomic impact but could also lead to additional consumer spending at the local scale.

## Supporting information

S1 File Supporting information document containing S1-S3 Figs and S1-S7 Tables. (DOCX)
