# Supplementary material for: Ambient air pollution and consumer spending: Evidence from Spain
Source: PLoS One. 2024 Jan 24;19(1):e0292245. doi: 10.1371/journal.pone.0292245 (PMC10807777; doi:10.1371/journal.pone.0292245)
Supplement: S1 File — (DOCX) [file pone.0292245.s001.docx]

**Ambient air pollution and consumer spending: Evidence from Spain**

**Supporting Information**

John Brandt^1^, Nihit Goyal^2^, Matthew Moroney^3^, Sophie Janaskie^4^, and Angel Hsu^5^

^1^ World Resources Institute, 10 G Street NE, Washington, DC 20002

^2^ Faculty of Technology, Policy and Management, TU Delft, Jaffalaan 5, 2628BX Delft, Netherlands

^3^ Raise Green, Inc, 444 Somerville Ave, MA 02143

^4^ Stanford Graduate School of Business, 655 Knight Way, Stanford, CA 94305

^5^ School of Public Policy, Abernethy Hall, 121 S. Columbia Street, Campus Box 3435, UNC-Chapel Hill, Chapel Hill, NC 27516

E-mail: nihit.goyal@tudelft.nl

**Fig S1. The distribution of O_3_, PM_2.5_, and spending in the municipalities included in the study.**

**Fig S2. Average consumer spending by age group per postal code by quarter.** Each point represents average spending for all study postal codes by day of the week per quarter, and bars highlight top and bottom quintiles of same-day ozone pollution.

**Fig S3. Average consumer spending by age group per postal code by quarter.** Each point represents average spending for all study postal codes by age group by day of the week per quarter, and bars highlight top and bottom quintiles of PM_2.5_ pollution.

**Table S1. Descriptive statistics for consumer spending (€) by age group.**

|  | **N** | **Mean** | **S.D.** |
| --- | --- | --- | --- |
|  |  |  |  |
| Age group: < 24 | 10391 | 1257.51 | 1911.32 |
| Age group: 25 - 34 | 10935 | 6936.23 | 7703.77 |
| Age group: 35 - 44 | 10938 | 10270.83 | 9910.44 |
| Age group: 45 - 54 | 10924 | 9017.73 | 12058.95 |
| Age group: 55 - 64 | 10853 | 5830.99 | 7347.43 |
| Age group: > 65 | 9840 | 3935.40 | 5410.92 |
|  |  |  |  |

**Table S2. The temporal displacement of consumer spending due to air pollution.**

|  | (1) | (2) | (3) | (4) | (5) | (6) |
| --- | --- | --- | --- | --- | --- | --- |
|  |  |  |  |  |  |  |
| O_3_ | -0.034* | -0.033+ | -0.035* | -0.031+ | -0.027+ | -0.027+ |
|  | [-0.067, -0.001] | [-0.066, 0.000] | [-0.069, 0.000] | [-0.064, 0.003] | [-0.060, 0.005] | [-0.059, 0.005] |
| O_3_, first lag | 0.008 | 0.021 | 0.024 | 0.021 | 0.018 | 0.016 |
|  | [-0.030, 0.047] | [-0.024, 0.067] | [-0.022, 0.070] | [-0.022, 0.065] | [-0.026, 0.063] | [-0.028, 0.060] |
| O_3_, second lag | - | -0.022 | -0.021 | -0.018 | -0.014 | -0.015 |
|  |  | [-0.049, 0.005] | [-0.049, 0.008] | [-0.045, 0.010] | [-0.043, 0.016] | [-0.045, 0.014] |
| O_3_, third lag | - | - | -0.003 | 0.001 | -0.001 | -0.002 |
|  |  |  | [-0.030, 0.024] | [-0.031, 0.034] | [-0.034, 0.032] | [-0.033, 0.030] |
| O_3_, fourth lag | - | - | - | -0.011 | 0.010 | 0.012 |
|  |  |  |  | [-0.046, 0.025] | [-0.032, 0.052] | [-0.027, 0.051] |
| O_3_, fifth lag | - | - | - | - | -0.036* | -0.031+ |
|  |  |  |  |  | [-0.066, -0.005] | [-0.063, 0.000] |
| O_3_, sixth lag | - | - | - | - | - | -0.007 |
|  |  |  |  |  |  | [-0.027, 0.013] |
| PM_2.5_ | 0.008 | 0.008 | 0.008 | 0.006 | 0.003 | 0.003 |
|  | [-0.007, 0.024] | [-0.007, 0.023] | [-0.008, 0.024] | [-0.010, 0.023] | [-0.014, 0.019] | [-0.013, 0.020] |
| PM_2.5_, first lag | -0.019+ | -0.020+ | -0.019+ | -0.017+ | -0.018+ | -0.020+ |
|  | [-0.041, 0.002] | [-0.040, 0.001] | [-0.039, 0.002] | [-0.036, 0.003] | [-0.038, 0.003] | [-0.041, 0.000] |
| PM_2.5_, second lag | - | 0.004 | 0.005 | 0.005 | 0.002 | 0.006 |
|  |  | [-0.012, 0.021] | [-0.012, 0.022] | [-0.013, 0.023] | [-0.017, 0.021] | [-0.013, 0.026] |
| PM_2.5_, third lag | - | - | -0.005 | -0.003 | -0.002 | -0.007 |
|  |  |  | [-0.024, 0.014] | [-0.023, 0.017] | [-0.022, 0.019] | [-0.028, 0.013] |
| PM_2.5_, fourth lag | - | - | - | -0.008 | -0.004 | -0.001 |
|  |  |  |  | [-0.025, 0.010] | [-0.021, 0.014] | [-0.018, 0.016] |
| PM_2.5_, fifth lag | - | - | - | - | -0.007 | -0.014 |
|  |  |  |  |  | [-0.025, 0.011] | [-0.033, 0.005] |
| PM_2.5_, sixth lag | - | - | - | - | - | 0.016+ |
|  |  |  |  |  |  | [-0.003, 0.035] |
| Temperature | -0.212* | -0.231* | -0.232* | -0.239* | -0.224* | -0.230* |
|  | [-0.382, -0.041] | [-0.409, -0.052] | [-0.409, -0.054] | [-0.419, -0.060] | [-0.408, -0.040] | [-0.420, -0.040] |
| Temperature, first lag | 0.087 | 0.078 | 0.063 | 0.090 | 0.078 | 0.100 |
|  | [-0.082, 0.257] | [-0.087, 0.242] | [-0.105, 0.231] | [-0.083, 0.262] | [-0.094, 0.251] | [-0.074, 0.275] |
| Temperature, second lag | - | 0.039 | 0.140 | 0.111 | 0.108 | 0.109 |
|  |  | [-0.139, 0.217] | [-0.048, 0.328] | [-0.090, 0.313] | [-0.091, 0.307] | [-0.087, 0.305] |
| Temperature, third lag | - | - | -0.102 | -0.070 | -0.071 | -0.080 |
|  |  |  | [-0.228, 0.023] | [-0.258, 0.118] | [-0.254, 0.112] | [-0.264, 0.104] |
| Temperature, fourth lag | - | - | - | -0.012 | 0.054 | 0.034 |
|  |  |  |  | [-0.142, 0.117] | [-0.099, 0.207] | [-0.115, 0.182] |
| Temperature, fifth lag | - | - | - | - | -0.077 | 0.052 |
|  |  |  |  |  | [-0.225, 0.072] | [-0.120, 0.223] |
| Temperature, sixth lag | - | - | - | - | - | -0.155* |
|  |  |  |  |  |  | [-0.286, -0.024] |
| Rain | 0.019 | 0.021+ | 0.024+ | 0.025+ | 0.020 | 0.020+ |
|  | [-0.005, 0.044] | [-0.004, 0.047] | [-0.002, 0.050] | [-0.002, 0.051] | [-0.004, 0.044] | [-0.004, 0.044] |
| Rain, first lag | -0.015 | -0.012 | -0.013 | -0.009 | -0.011 | -0.014 |
|  | [-0.034, 0.004] | [-0.032, 0.007] | [-0.032, 0.006] | [-0.027, 0.009] | [-0.029, 0.006] | [-0.035, 0.007] |
| Rain, second lag | - | -0.010 | -0.007 | -0.008 | -0.006 | -0.008 |
|  |  | [-0.027, 0.006] | [-0.025, 0.011] | [-0.026, 0.010] | [-0.024, 0.012] | [-0.026, 0.010] |
| Rain, third lag | - | - | -0.003 | -0.002 | -0.002 | 0.000 |
|  |  |  | [-0.026, 0.019] | [-0.026, 0.021] | [-0.025, 0.021] | [-0.021, 0.021] |
| Rain, fourth lag | - | - | - | -0.005 | 0.002 | 0.003 |
|  |  |  |  | [-0.028, 0.017] | [-0.019, 0.022] | [-0.017, 0.023] |
| Rain, fifth lag | - | - | - | - | -0.007 | 0.003 |
|  |  |  |  |  | [-0.026, 0.012] | [-0.015, 0.021] |
| Rain, sixth lag | - | - | - | - | - | -0.012 |
|  |  |  |  |  |  | [-0.032, 0.007] |
| Pressure | -0.182 | -0.211 | -0.234 | -0.168 | -0.151 | -0.174 |
|  | [-0.740, 0.375] | [-0.785, 0.363] | [-0.822, 0.355] | [-0.756, 0.421] | [-0.735, 0.434] | [-0.802, 0.453] |
| Pressure, first lag | -0.072 | -0.161 | -0.201 | -0.165 | -0.289 | -0.187 |
|  | [-0.685, 0.541] | [-0.864, 0.542] | [-0.902, 0.499] | [-0.887, 0.557] | [-1.019, 0.442] | [-0.941, 0.567] |
| Pressure, second lag | - | 0.262 | 0.532 | 0.539 | 0.566 | 0.523 |
|  |  | [-0.419, 0.943] | [-0.290, 1.353] | [-0.333, 1.410] | [-0.309, 1.441] | [-0.352, 1.399] |
| Pressure, third lag | - | - | -0.276 | -0.260 | -0.321 | -0.345 |
|  |  |  | [-0.949, 0.397] | [-1.111, 0.592] | [-1.203, 0.560] | [-1.250, 0.560] |
| Pressure, fourth lag | - | - | - | -0.009 | 0.304 | 0.231 |
|  |  |  |  | [-0.615, 0.596] | [-0.541, 1.149] | [-0.656, 1.119] |
| Pressure, fifth lag | - | - | - | - | -0.250 | 0.181 |
|  |  |  |  |  | [-1.004, 0.504] | [-0.677, 1.039] |
| Pressure, sixth lag | - | - | - | - | - | -0.447 |
|  |  |  |  |  |  | [-1.126, 0.233] |
| Dewpoint temperature | -0.002 | 0.008 | 0.003 | -0.010 | 0.002 | 0.001 |
|  | [-0.059, 0.056] | [-0.046, 0.062] | [-0.049, 0.055] | [-0.061, 0.042] | [-0.049, 0.053] | [-0.049, 0.050] |
| Dewpoint temperature, first lag | 0.102** | 0.073* | 0.066+ | 0.065+ | 0.071* | 0.077* |
|  | [0.038, 0.167] | [0.003, 0.142] | [-0.004, 0.135] | [-0.006, 0.136] | [0.001, 0.141] | [0.005, 0.149] |
| Dewpoint temperature, second lag | - | 0.030 | 0.025 | 0.020 | 0.015 | 0.015 |
|  |  | [-0.043, 0.104] | [-0.054, 0.103] | [-0.063, 0.102] | [-0.069, 0.098] | [-0.069, 0.099] |
| Dewpoint temperature, third lag | - | - | 0.012 | -0.021 | -0.018 | -0.022 |
|  |  |  | [-0.055, 0.080] | [-0.086, 0.044] | [-0.087, 0.052] | [-0.090, 0.045] |
| Dewpoint temperature, fourth lag | - | - | - | 0.071+ | 0.084* | 0.078* |
|  |  |  |  | [0.000, 0.142] | [0.006, 0.161] | [0.002, 0.154] |
| Dewpoint temperature, fifth lag | - | - | - | - | -0.051 | -0.053 |
|  |  |  |  |  | [-0.130, 0.028] | [-0.122, 0.016] |
| Dewpoint temperature, sixth lag | - | - | - | - | - | -0.016 |
|  |  |  |  |  |  | [-0.077, 0.045] |
|  |  |  |  |  |  |  |
| N | 8473 | 8336 | 8217 | 8116 | 8023 | 7938 |
| R^2^ | 0.928 | 0.929 | 0.929 | 0.929 | 0.930 | 0.930 |
| R^2^ Adj. | 0.924 | 0.924 | 0.925 | 0.925 | 0.925 | 0.926 |

The unit of analysis is postal code with daily frequency. The dependent variable is the log of consumer spending. The independent variables and control variables have been normalized. The regressions include postal code fixed effect, date fixed effect, and monthly trend by postal code. The standard errors in brackets are clustered by postal code and date. + p < 0.1, * p < 0.05, ** p < 0.01, *** p < 0.001.

**Table S3. The regression of consumer spending on one-day prior air pollution.**

|  | (1) | (2) | (3) | (4) |
| --- | --- | --- | --- | --- |
|  |  |  |  |  |
| O_3_, first lag | -0.033 | - | -0.035+ | -0.010 |
|  | [-0.073, 0.007] |  | [-0.075, 0.005] | [-0.045, 0.025] |
| PM_2.5_, first lag | - | -0.021* | -0.023* | -0.019+ |
|  |  | [-0.041, -0.001] | [-0.044, -0.002] | [-0.040, 0.003] |
| Temperature | - | - | - | -0.158* |
|  |  |  |  | [-0.294, -0.022] |
| Rain | - | - | - | 0.018 |
|  |  |  |  | [-0.005, 0.042] |
| Pressure | - | - | - | -0.217 |
|  |  |  |  | [-0.779, 0.344] |
| Dewpoint temperature | - | - | - | 0.056+ |
|  |  |  |  | [-0.011, 0.124] |
|  |  |  |  |  |
| N | 8408 | 8408 | 8408 | 8408 |
| R^2^ | 0.925 | 0.925 | 0.925 | 0.927 |
| R^2^ Adj. | 0.921 | 0.921 | 0.921 | 0.923 |

The unit of analysis is postal code with daily frequency. The dependent variable is the log of consumer spending. The independent variables and control variables have been normalized. The regressions include postal code fixed effect, date fixed effect, and monthly trend by postal code. The standard errors in brackets are clustered by postal code and date. + p < 0.1, * p < 0.05, ** p < 0.01, *** p < 0.001.

**Table S4. Robustness check on effect of variation in functional form of air pollution.**

|  | (1) | (2) | (3) |
| --- | --- | --- | --- |
|  |  |  |  |
| Log (O_3_) | -0.090** | - | - |
|  | [-0.144, -0.036] |  |  |
| O_3_ | - | -0.041** | - |
|  |  | [-0.069, -0.014] |  |
| Square (O_3_) | - | 0.009 | - |
|  |  | [-0.003, 0.021] |  |
| Spline 1: O_3_ | - | - | -0.152 |
|  |  |  | [-0.402, 0.098] |
| Spline 2: O_3_ | - | - | -0.270** |
|  |  |  | [-0.468, -0.072] |
| Spline 3: O_3_ | - | - | -0.178 |
|  |  |  | [-0.404, 0.049] |
| Log (PM_2.5_) | -0.006 | - | - |
|  | [-0.038, 0.026] |  |  |
| PM_2.5_ | - | -0.007 | - |
|  |  | [-0.027, 0.013] |  |
| Square (PM_2.5_) | - | 0.004+ | - |
|  |  | [0.000, 0.009] |  |
| Spline 1: PM_2.5_ | - | - | -0.162 |
|  |  |  | [-0.386, 0.062] |
| Spline 2: PM_2.5_ | - | - | 0.180 |
|  |  |  | [-0.114, 0.474] |
| Spline 3: PM_2.5_ | - | - | 0.053 |
|  |  |  | [-0.220, 0.327] |
| Temperature | -0.115* | -0.116* | -0.117* |
|  | [-0.212, -0.019] | [-0.213, -0.019] | [-0.214, -0.020] |
| Rain | 0.012 | 0.012 | 0.012 |
|  | [-0.003, 0.027] | [-0.003, 0.027] | [-0.004, 0.027] |
| Pressure | -0.079 | -0.086 | -0.084 |
|  | [-0.444, 0.286] | [-0.450, 0.278] | [-0.448, 0.280] |
| Dewpoint temperature | 0.056* | 0.056* | 0.056* |
|  | [0.002, 0.110] | [0.002, 0.109] | [0.003, 0.110] |
|  |  |  |  |
| N | 10948 | 10948 | 10948 |
| R^2^ | 0.921 | 0.921 | 0.921 |
| R^2^ Adj. | 0.917 | 0.917 | 0.917 |

The unit of analysis is postal code with daily frequency. The dependent variable is the log of consumer spending. The independent variables and control variables have been normalized. The regressions include postal code fixed effect, date fixed effect, and monthly trend by postal code. The standard errors in brackets are clustered by postal code and date. + p < 0.1, * p < 0.05, ** p < 0.01, *** p < 0.001.

**Table S5. Robustness check on variation in fixed effect.**

|  | (1) | (2) | (3) | (4) | (5) |
| --- | --- | --- | --- | --- | --- |
|  |  |  |  |  |  |
| O_3_ | -0.054+ | -0.042** | -0.095** | -0.017 | -0.044* |
|  | [-0.108, 0.001] | [-0.071, -0.013] | [-0.153, -0.038] | [-0.046, 0.013] | [-0.081, -0.007] |
| PM_2.5_ | 0.029 | 0.004 | 0.029 | 0.014 | 0.011 |
|  | [-0.016, 0.075] | [-0.009, 0.018] | [-0.016, 0.074] | [-0.007, 0.034] | [-0.006, 0.029] |
| Temperature | 0.027 | -0.110* | -0.015 | -0.006 | -0.008 |
|  | [-0.034, 0.089] | [-0.196, -0.024] | [-0.133, 0.103] | [-0.046, 0.034] | [-0.081, 0.065] |
| Rain | 0.018 | 0.009 | 0.014 | 0.022* | 0.022* |
|  | [-0.009, 0.045] | [-0.006, 0.024] | [-0.012, 0.040] | [0.003, 0.041] | [0.002, 0.043] |
| Pressure | 0.042 | -0.149 | -0.020 | -0.010 | -0.070 |
|  | [-0.336, 0.420] | [-0.494, 0.197] | [-0.420, 0.380] | [-0.179, 0.158] | [-0.272, 0.131] |
| Dewpoint temperature | 0.017 | 0.062* | 0.046 | 0.073*** | 0.085*** |
|  | [-0.048, 0.083] | [0.000, 0.124] | [-0.025, 0.118] | [0.031, 0.116] | [0.043, 0.127] |
|  |  |  |  |  |  |
| Postal code fixed effect | X | X | X | X | X |
| Postal code specific monthly trend | X |  |  |  | X |
| Date fixed effect |  | X |  |  |  |
| Month fixed effect |  |  | X |  | X |
| Day of week fixed effect |  |  |  | X | X |
|  |  |  |  |  |  |
| N | 10948 | 10948 | 10948 | 10948 | 10948 |
| R^2^ | 0.733 | 0.917 | 0.736 | 0.858 | 0.867 |
| R^2^ Adj. | 0.729 | 0.914 | 0.733 | 0.857 | 0.865 |
|  | | |  |  |  |

The unit of analysis is postal code with daily frequency. The dependent variable is the log of consumer spending. The independent variables and control variables have been normalized. The standard errors in brackets are clustered by postal code and date. + p < 0.1, * p < 0.05, ** p < 0.01, *** p < 0.001.

**Table S6. Variation in the association of air pollution with consumer spending based on distance from monitoring station.**

|  | Nearby station | Distant station |
| --- | --- | --- |
|  |  |  |
| O_3_ | -0.040** | -0.039 |
|  | [-0.069, -0.011] | [-0.101, 0.022] |
| PM_2.5_ | 0.002 | 0.010 |
|  | [-0.012, 0.017] | [-0.009, 0.029] |
| Temperature | -0.128* | -0.099 |
|  | [-0.230, -0.026] | [-0.318, 0.120] |
| Rain | 0.014+ | 0.001 |
|  | [-0.002, 0.030] | [-0.032, 0.034] |
| Pressure | -0.156 | 0.337 |
|  | [-0.548, 0.236] | [-0.404, 1.078] |
| Dewpoint temperature | 0.056+ | 0.101+ |
|  | [0.000, 0.113] | [-0.017, 0.220] |
|  |  |  |
| N | 9069 | 1879 |
| R^2^ | 0.927 | 0.871 |
| R^2^ Adj. | 0.922 | 0.836 |

A nearby station is one that is located at a distance of 10 km or less from the centroid of the postal code. The unit of analysis is postal code with daily frequency. The dependent variable is the log of consumer spending. The independent variables and control variables have been normalized. The regressions include postal code fixed effect, date fixed effect, and monthly trend by postal code. The standard errors in brackets are clustered by O_3_ monitoring station, PM_2.5_ monitoring station, and date. + p < 0.1, * p < 0.05, ** p < 0.01, *** p < 0.001.

**Table S7. Variation in the association of air pollution with consumer spending based on average pollution level.**

|  | Low O_3_ | Low PM_2.5_ |
| --- | --- | --- |
|  |  |  |
| O_3_ | -0.034 | -0.053* |
|  | [-0.078, 0.010] | [-0.092, -0.013] |
| PM_2.5_ | -0.004 | 0.000 |
|  | [-0.031, 0.023] | [-0.024, 0.024] |
| Temperature | -0.051 | -0.189** |
|  | [-0.143, 0.041] | [-0.324, -0.054] |
| Rain | 0.023+ | 0.010 |
|  | [-0.002, 0.047] | [-0.012, 0.032] |
| Pressure | 0.083 | -0.167 |
|  | [-0.330, 0.496] | [-0.670, 0.337] |
| Dewpoint temperature | -0.007 | 0.068+ |
|  | [-0.060, 0.045] | [-0.003, 0.140] |
|  |  |  |
| N | 2527 | 5052 |
| R^2^ | 0.952 | 0.907 |
| R^2^ Adj. | 0.943 | 0.897 |

Low O_3_ is defined as O_3_ concentration less than 50 µg/m^3^ while low PM_2.5_ is defined as PM_2.5_ concentration less than 10 µg/m^3^. The unit of analysis is postal code with daily frequency. The dependent variable is the log of consumer spending. The independent variables and control variables have been normalized. The regressions include postal code fixed effect, date fixed effect, and monthly trend by postal code. The standard errors in brackets are clustered by O_3_ monitoring station, PM_2.5_ monitoring station, and date. + p < 0.1, * p < 0.05, ** p < 0.01, *** p < 0.001.
